# A review of pathological findings in impalas (*Aepyceros melampus*) in South Africa

**DOI:** 10.4102/jsava.v91i0.1965

**Published:** 2020-08-11

**Authors:** Caroline Chu, Johan Steyl, Elizabeth C. du Plessis, Bjorn Reininghaus, Emily P. Mitchell

**Affiliations:** 1 Zoological Pathology Program, University of Illinois, Chicago, United States; 2 Charles River Laboratories, Senneville, Canada; 3 Department of Paraclinical Sciences, Faculty of Veterinary Science, University of Pretoria, Onderstepoort, South Africa; 4 IDEXX Laboratories, Derdepoortpark, Pretoria, South Africa; 5 Mpumulanga Veterinary Services, Nelspruit, South Africa; 6 Department of Paraclinical Sciences, Faculty of Veterinary Science, University of Pretoria, Pretoria, South Africa; 7 National Zoological Garden, South African National Biodiversity Institute, Pretoria, South Africa

**Keywords:** *Aepyceros melampus*, disease, impala, parasites, pathology

## Abstract

Impalas (*Aepyceros melampus*) are common African antelope. A retrospective study was conducted of 251 impala cases from game farms, national parks and zoos submitted by veterinarians and pathologists in South Africa (2003–2016). Histopathology slides as well as records of macroscopic lesions and additional diagnostic tests performed were examined. Non-infectious conditions, such as acute pulmonary congestion and oedema, cachexia, traumatic injury and anaesthetic-related mortality were the most common causes of morbidity and mortality. Bacterial sepsis was the most common infectious disease, whilst skeletal muscle and myocardial sarcocystosis and verminous cholangitis and pneumonia were the most common parasitic diseases. Although the retrospective nature of this study limits the significance of the relative prevalence of lesions in the three locations, management decisions and diagnostic plans may be informed by the results. Impala from game farms had significantly more cachexia cases than those from other locations. Impala from zoos had significantly more lymphoid depletion than those from other locations. These findings suggest that nutrition and pasture management, enclosure design, management of intra- and interspecies aggression and improved anaesthetic protocols could improve animal welfare and survival of impala on game farms and in zoos. This report presents a detailed survey of diseases and conditions found in impala that provides baseline data for veterinary pathologists.

## Introduction

The impala (*Aepyceros melampus*) is a medium-sized antelope distributed widely throughout the woodlands and savannahs of East, Central and Southern Africa. They typically select areas with nutrient-rich soils providing good-quality grass during the wet season and high-quality browsing consisting of leaves and fine twigs, in the dry season (Blanchard & Fritz 2008; Bothma, Van Rooyen & Du Toit 2016).

The current South African impala population is estimated at almost two million, of which approximately 50% are on private land and 25% in protected areas including the Kruger National Park (KNP) in South Africa (Fritz & Bourgarel 2013; International Union for Conservation of Nature Species Survival Commission [IUCN SSC] Antelope Specialist Group 2016). Although previously eliminated from many areas, impalas are now widespread and abundant on privately owned game farms, which have markedly variable management, species mixtures and stocking densities. They are an important commercial game hunting species and a source of meat for private consumption in South Africa (Bothma et al. 2016). Coat colour variant traits were artificially promoted in the past amongst game farms, through intensive selective breeding, as these individuals commanded premium prices. Small numbers of impalas are housed in South African zoological institutions primarily for educational purposes, often as part of mixed African savannah exhibits. The National Zoological Garden (NZG) of the South African National Biodiversity Institute has a small population of black-faced impalas (*A. melampus petersi*), a darker and larger subspecies from north-western Namibia and south-western Angola.

Apart from extensive literature on the life history, ecology, behaviour, genetics, anaesthesia, biochemistry and physiology of impalas, the presence of ectoparasites (*Gedoelstia* and various tick species), protozoa (besnoitia, coccidia, hepatozoon and sarcocystis), various helminths, pentastomes, and bacterial (including anthrax, brucellosis and tuberculosis) and viral (foot and mouth disease and lumpy skin disease) infections have been recorded in impalas (Abu Samra et al. 2013; Bengis 2000; Boomker et al. 2000; Bryden & De Vos 1994; Gallivan & Surgeoner 1995; Gallivan et al. 1989, 1996; Horak et al. 2003; Karesh et al. 1997; Lane 1995; Loomis & Wright 1989; Okori 1999; Pletcher et al. 1988). Impalas in the KNP are highly susceptible to anthrax (De Vos & Turnbull 2004; Gates, Elkin & Dragon 2001), and outbreaks of foot and mouth disease (FMD) have been regularly reported in impalas in the KNP over the last 60 years (Thomson & Bastos 2004). However, less information is available on the relationship between the presence of the pathogen, disease susceptibility and causes of mortality, particularly in impalas from game farms (GFs). The aim of this study was to document lesions in impala tissues submitted to South African pathologists, and to provide baseline information on disease and lesion prevalence in different management systems that veterinarians and veterinary pathologists can use to inform management and treatment plans, necropsy sampling strategies and diagnoses.

## Research methods and design

A retrospective review of 251 impala cases, submitted to the Faculty of Veterinary Science at the University of Pretoria (135/251), the NZG (70/251) and IDEXX Laboratories (31/251), for pathological evaluation between 2003 and 2016, was performed. Macroscopic necropsy and histopathology reports, records of additional diagnostic tests performed and archived histopathology slides were examined. Impalas from GFs had variable access to supplemental nutrition for at least part of the year and were variably used for hunting or meat production. Impalas from national parks (NPs) were mostly from the Greater KNP and restricted tissue sets were submitted primarily for surveillance of tuberculosis. They did not receive supplemental nutrition or other management interventions. Impalas housed in zoos received various diets and were used for research, conservation or public display.

Full tissue sets were not available for all animals. Tissues submitted for histopathology were fixed in 10% neutral buffered formalin, processed routinely through graded alcohols and embedded in paraffin wax. Histologic sections were prepared using haematoxylin and eosin (H&E), Ziehl-Neelsen and Gram stains. Age, sex, location, macroscopic and histological lesions, and the results of any supporting diagnostic tests that were performed (immunohistochemistry, microbiology, molecular diagnostic) were reviewed and collated by a single pathologist (Caroline Chu). Each case was assigned a final diagnosis based on the major disease process and cause of death or reason for euthanasia. Morphological diagnoses were assigned for individual lesions in each major organ system and the proportion of impalas with each lesion was calculated on the number of cases for which the relevant tissue was sampled. Due to the small numbers of black-faced impalas (*n* = 13) and coat colour variants (*n* = 14), these animals were not analysed separately. Age categories were not rigorously defined by the referring veterinarian or owner. Many cases had no information available regarding sex (*n* = 83, 33%) and/or age (*n* = 74, 30%) and, therefore, the effect of these parameters on disease or lesion prevalence was not evaluated. Disease and lesion prevalence were compared between impalas from the three locations using Fisher’s exact test. *P*-values less than 0.05 were considered statistically significant.

### Ethical consideration

The study was approved by the NZG Research and Ethics Committee (P06/25).

## Results

Of the 251 impalas, 151 were from GFs (60%), 83 from NPs (33%) and 14 (6%) from zoos, whilst the location of three animals (1%) was not provided (Table 1). In those animals for which information was provided, approximately equal proportions of male (37%) and female (30%) were sampled, and most of the impalas were adult (43%), normal coloured (84%) common impalas (95%). The locations of impalas with non-infectious conditions are listed in Table 2. The locations of impalas with parasitic and infectious or inflammatory diseases are listed in Table 3. In a relatively high proportion of cases (14%, 34/251) a cause of death could not be determined.

**TABLE 1:** Location, age, sex, coat colour and subspecies of 251 impalas (*Aepyceros melampus*).

Parameter	Location	Number	%
Location	Game farm	151	60
	National park	83	33
	Zoo	14	6
	Unknown	3	1
Sex	Male	93	37
	Female	75	30
	Unknown	83	33
Age†	Adult	108	43
	Subadult	28	11
	Juvenile	26	10
	Foetal	2	1
	Unknown	74	30
Coat colour	Normal	210	84
	Black	35	14
	Split‡	3	1
	White	3	1
Subspecies	*A. m. melampus*	238	95
	*A. m. petersi*	13	5

† , Age estimated by owner or referring veterinarian.

‡ , Impalas with heterogenous genes for black coat colour.

**TABLE 2:** Location of impalas (*Aepyceros melampus*) with non-infectious conditions.

Condition	Affected impala (location)†							
	Game farm		National park		Zoo		Total	
	*n*	%	*n*	%	*n*	%	*n*	%
Pulmonary congestion, oedema* (*n* = 200)	84	63	20	40	8	62	113	57
Cachexia* (*n* = 251)	60	40	7	8	1	7	68	27
Renal tubular mineralisation (*n* = 187)	32	24	4	11	2	14	39	21
Traumatic muscle injury (*n* = 144)	18	18	6	15	6	46	30	20
Testicular atrophy (*n* = 16)	3	33	0	-	0	-	3	19
Adrenocortical hyperplasia (*n* = 51)	2	9	5	33	2	18	9	18
Skeletal muscle degeneration, necrosis (*n* = 144)	15	15	2	6	2	17	19	13
Terminal aspiration rumen fluid (*n* = 200)	14	10	7	12	7	12	24	12
Myocardial degeneration, necrosis (*n* = 185)	16	12	2	5	3	23	21	11
Myoglobinuric nephrosis (*n* = 187)	12	9	0	-	1	7	13	7
Hepatic degeneration, necrosis (*n* = 198)	11	8	2	4	0	-	13	6
Myocardial fibrosis (*n* = 185)	9	7	1	2	2	15	12	6
Lymphoid depletion* (*n* = 193)	5	4	2	3	4	31	11	6
Ruminal mineralisation** (*n* = 115)	1	1	2	13	2	14	5	4
Ruminal acidosis (*n* = 115)	2	2	0	-	1	7	3	3
Hepatocellular nuclear vacuoles (*n* = 198)	6	5	0	-	0	-	6	3
Myocardial, endocardial mineralisation (*n* = 185)	3	2	2	5	0	-	5	3
Neuronal necrosis (*n* = 109)	1	1	1	6	0	-	2	2

† , The proportion of impalas with each lesion was calculated on the number of animals at each location for which the relevant tissue was available. The location of three impalas was not known.

* , Statistically significant (*p* < 0.05) differences between impala from different locations, *p* < 0.05.

**TABLE 3:** Location of impalas (*Aepyceros melampus*) with common parasitic, infectious and inflammatory diseases.

Condition	Lesion	Affected impala (location)†							
		Game farm		National park		Zoo		Total	
		*n*	%	*n*	%	*n*	%	*n*	%
Parasitic	Muscle sarcocystosis* (*n* = 144)	47	48	10	29	4	33	61	42
	Verminous cholangitis* (*n* = 198)	27	20	21	43	3	21	52	26
	Myocardial sarcocystosis (*n* = 185)	24	18	5	12	2	15	32	17
	Verminous pneumonia* (*n* = 200)	8	6	18	36	0	-	26	13
	Ectoparasitic dermatitis (*n* = 49)	0	-	5	?	0	-	5	-
	Intestinal coccidiosis (*n* = 115)	8	10	0	-	0	-	8	7
	Theileriosis (*n* = 251)	4	3	0	-	0	-	4	2
Infectious/inflammatory	Enteritis (*n* = 115)	28	34	3	13	4	29	36	31
	Portal hepatitis (*n* = 198)	29	22	8	14	2	14	41	21
	Endometritis (*n* = 13)	1	14	0		1	20	2	15
	Myocarditis (*n* = 185)	17	13	7	16	1	8	25	13
	Interstitial nephritis (*n* = 187)	9	7	4	11	1	7	15	8
	Bacterial sepsis (*n* = 251)	12	8	3	4	2	14	17	7
	Dermatitis (*n* = 49)	4	25	9	35	0	-	13	7
	Cutaneous papilloma (*n* = 49)	0	-	2	8	0	-	2	4
	Bacterial pneumonia (*n* = 200)	2	1	4	7	0	-	6	3
	Necrobacillosis (*n* = 251)	3	2	0	-	0	-	3	1

† , The proportion of impalas with each lesion was calculated on the number of animals at each location for which the relevant tissue was available. The location of three impalas was not known.

* , Statistically significant (*p* < 0.05) differences between impala from different locations.

### Non-infectious conditions

#### Pulmonary congestion and oedema

Acute diffuse pulmonary congestion and oedema was the most common respiratory system lesion and the most common change observed in all cases (57%, 113/200), occasionally accompanied by acute widespread tissue congestion. Thirty-three (17%) animals also had regional to diffuse atelectasis, as well as multiple associated foci of acute pulmonary haemorrhage (interpreted as agonal diapedesis). These changes were observed significantly (*p* = 0.02) more commonly in impalas from GFs (63%, 84/134) and zoos (62%, 8/13) than in animals from NPs (40%, 20/50).

#### Cachexia

Impalas with variable combinations of macroscopic or histological evidence of poor body condition, serous atrophy of renal, cardiac and bone marrow adipose tissue, pancreatic zymogen granule depletion, anaemia and liver, spleen, ruminal mucosa, skeletal muscle and testicular atrophy were diagnosed as cachexic. Cachexia was diagnosed in 27% of cases (68/251). Significantly (*p* = 0.003) more impalas from GFs were cachexic (40%, 60/151) compared to those from NPs (8%, 7/83) or zoos (7%, 1/14). Cachexia was often accompanied by one or more additional non-infectious conditions (including adrenocortical hyperplasia [*n* = 8], dehydration [*n* = 6], traumatic injury [*n* = 5], suspected toxic liver disease [*n* = 4], parathyroid gland hyperplasia [*n* = 2], rumen acidosis [*n* = 1]), or infectious/parasitic diseases (including coccidiosis [*n* = 7], bacterial infection [*n* = 5], internal parasites [*n* = 4], lung worm infestation [*n* = 3], theileriosis [*n* = 3], pneumonia [*n* = 3] and viral enteritis [*n* = 1]). One animal had both coccidia and theileriosis, and another had coccidia and myopathy. Two cachexic lambs from zoos had not suckled before death. Of the impalas that died during or after capture, 19% (8/42) were cachexic. One cachexic animal that died during anaesthesia also had lungworm. Seven cachexic animals had been translocated in the weeks prior to death. Cachexia, with no history of capture, was present in four impalas.

#### Traumatic injury

Skeletal fractures with associated muscular haemorrhage, joint luxations, cutaneous lacerations and puncture wounds were more common in impalas from zoos (46%, 6/13) than in those from GFs (18%, 18/99) or NPs (15%, 6/40), but this difference was not significant (*p* = 0.054). Many of these animals were found dead or subsequently euthanised due to their injuries. Details of the circumstances in which traumatic injuries occurred were rarely supplied, but five cases were due to intra- or interspecies aggression; four were associated with recent capture; three impalas were caught in a fence; two were trampled neonates (one each from a NP and a zoo) and one from a NP was hit by a car.

#### Myopathy

Skeletal muscle (13%, 19/144) and myocardial (11%, 21/185) myofibre degeneration and/or necrosis occurred in impalas from all three locations, often in the same animal. Acute skeletal muscle necrosis occurred in recently captured animals (*n* = 14), those that died of cachexia (*n* = 9) and in two recently captured cachexic impalas. Necrotic cardiac myocytes in a black-faced impala from a zoo that had not been captured showed more long-standing multifocal muscle regeneration with reactive satellite cells and small numbers of neutrophils and macrophages. Twelve impalas from GFs and one from a zoo (7%, 13/187) with myopathy had acute renal tubular degeneration and/or necrosis associated with intraluminal accumulations of red granular pigment consistent with myoglobin.

#### Lymphoid atrophy

Eleven impalas had decreased numbers of lymphocytes with loss of cortical medullary distinction and/or follicular architecture in the lymph nodes, spleen and/or thymus. Lymphoid depletion was significantly (*p* = 0.006) more common in impalas from zoos (31%, 4/13) than in those from GFs (4%, 5/116) or NPs (3%, 2/61). Affected animals from NPs and zoos were mostly neonates, in which case lymphoid hypoplasia could not be distinguished from lymphoid atrophy.

#### Miscellaneous non-infectious conditions

In six impalas from GFs and two from NPs (4%, 8/198), centrilobular to massive hepatocellular degeneration, necrosis and haemorrhage were present with occasional hepatocellular megalocytosis and canalicular bile stasis (consistent with toxic liver damage). One impala, from a GF, had concurrent ulcerative dermatitis of the external pinna. One impala from a GF had scattered individual cerebrocortical and Purkinje cell neuronal necrosis with small accumulations of golden-brown, finely granular cytoplasmic pigment in glial cells and viable neurons and spheroids within white matter (consistent with toxic neuropathy).

The rumens of two animals from GFs and one from a zoo had multiple intraepithelial pustules with or without epithelial hyperplasia and orthokeratotic hyperkeratosis, consistent with ruminal acidosis. Although uncommon, multifocal dystrophic mineralisation of the rumen mucosa was significantly more common (*p* = 0.02) in animals from zoos (14%, 2/14) than in those from GFs (1%, 1/83) or NPs (13%, 2/16).

**FIGURE 1:**
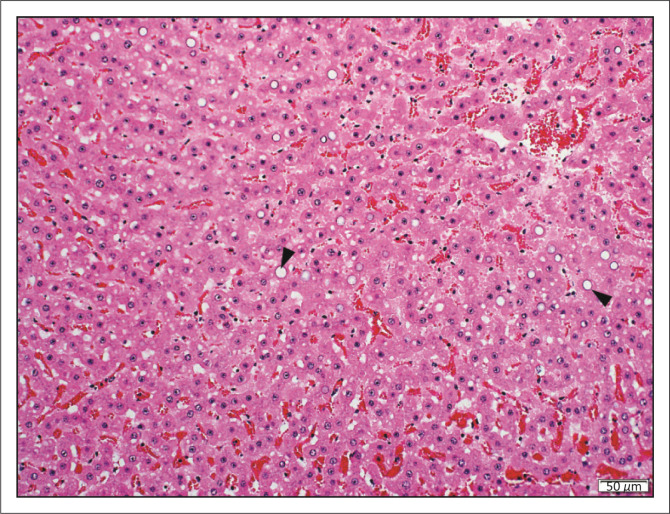
Hepatocyte nuclear vacuolar degeneration. A few midzonal to centrilobular hepatocytes have enlarged nuclei up to 25 *μ*m in diameter with a large distinct, central, colourless vacuole (arrowheads) (haematoxylin and eosin [H&E]).

Nine impalas (18%, 9/63) showed diffuse adrenal cortical hyperplasia. Twelve (6%, 12/185) impalas had multifocal mild interstitial to perivascular myocardial fibrosis. Five animals (3%, 5/185) had multifocal myocardial and/or endocardial mineralisation. Keratin flakes and meconium were observed in alveoli of three neonatal impalas, consistent with meconium aspiration, indicative of foetal distress during parturition. Six impalas from GFs (3%, 6/198) had unusual centrilobular to midzonal hepatocellular nuclear vacuolation. Nuclei were typically swollen up to 25 *µ*m in diameter with a single large, colourless, distinct, central vacuole (Figure 1). Small numbers of affected nuclei had pale eosinophilic, homogenous pseudoinclusions. Mild multifocal renal tubular mineralisation was present in 21% (39/187) of cases. One elderly black-faced impala had concurrent uterine adenocarcinoma and cystic endometrial hyperplasia. One impala from a GF had an adrenal gland pheochromocytoma. One black-faced impala, which was euthanised due to declining body condition, had one small focus of bronchioloalveolar carcinoma in one lung lobe. An adult male from a NP had a serosal mesothelioma.

Twenty-four (12%, 24/200) impalas from all three locations showed mild, likely peri-mortem, aspiration of ruminal contents. In 26 (14%, 26/94) impalas, erythrocytes and fibrin within lymph node medullary sinuses were present, consistent with agonal draining of areas of haemorrhage.

### Parasitic diseases

Sarcocystosis was the most common parasitic condition recorded as well as the most common histological lesion in skeletal muscle and myocardium. Skeletal muscle (42%, 61/144) and myocardium (17%, 32/185) of impalas from all three locations had protozoal cysts compatible with *Sarcocystis* sp. The cysts were not associated with inflammation or myocyte damage, apart from an adult female impala purchased from an auction 8 days previously, which was in poor condition and had worn teeth. Lymphohistiocytic myocarditis associated with *Sarcocystis* cysts was thought to have contributed to the death of this animal.

**FIGURE 2:**
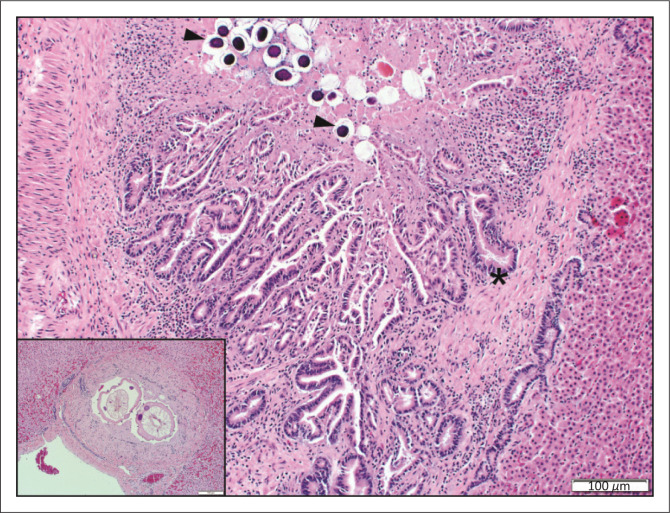
Verminous cholangitis. Hyperplastic bile duct epithelium (*) surrounded by eosinophilic and granulomatous to lymphocytic inflammation with intraluminal nematode eggs (arrowheads) (haematoxylin and eosin [H&E]). Inset: Spiny *Cooperioides hepaticae* in a bile duct with extensive mural fibrosis.

The most common hepatic lesion was chronic-active proliferative, occasionally necrotising, eosinophilic and granulomatous verminous cholangitis (26%, 52/198) that frequently extended into the surrounding portal area, and in severe cases, completely obscured portal architecture (Figure 2). Twelve (23%) of the cholangitis cases were associated with intraluminal adult filarial nematodes and embryonated ova consistent with *Cooperioides hepaticae*. Verminous cholangitis was significantly more common (*p* = 0.01) in impalas from NPs (43%, 21/49) than in impalas from zoos (21%, 3/14) or GFs (20%, 27/132).

**FIGURE 3:**
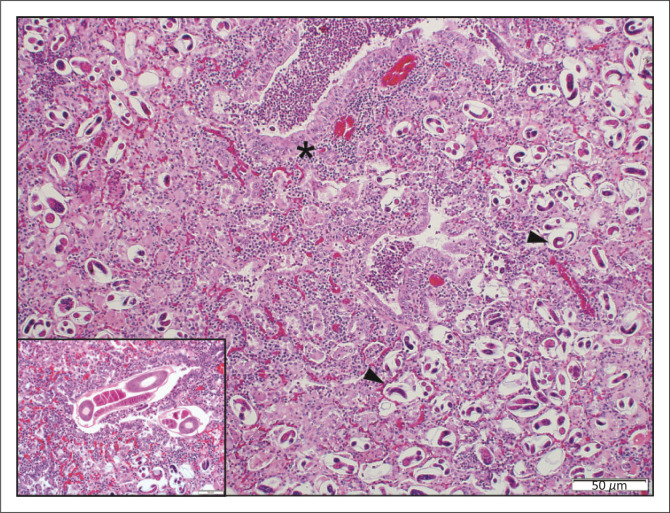
Verminous pneumonia. Myriad microfilaria (arrowheads) and a mixed inflammatory response obscure pulmonary architecture. Bronchiolar epithelium is hyperplastic (*) and bronchiolar lumina filled with degenerating neutrophils (haematoxylin and eosin [H&E]). Inset: Adult *Pneumostrongylus calcaratus* nematodes (H&E).

Eight impalas from GFs and 18 from NPs had chronic-active eosinophilic interstitial and granulomatous pneumonia with intralesional adult metastrongyle nematodes, larvae and embryonated ova that were morphologically consistent with *Pneumostrongylus calcaratus* (Figure 3). Larger granulomas appeared to be histologically similar to tuberculosis granulomas, although no acid-fast bacilli were observed within the lesions. Verminous pneumonia was significantly (*p* < 0.0001) more common in impalas from NPs (36%, 18/50) than in those from GFs (6%, 8/134). In eight impalas (9%, 8/89), intestinal intra luminal nematodes and/or cestodes were noted macroscopically or histologically, with minimal associated inflammation.

Five impalas from NPs had eosinophilic and lymphoplasmacytic dermatitis with epithelial hyperplasia and hyperkeratosis. Three of these animals had intra-follicular arthropods morphologically consistent with *Demodex* sp. mites or lice such as *Damalinia* sp. or *Linognathus* spp. One of these impalas also had large serocellular crusts with intralesional bacterial cocci.

**FIGURE 4:**
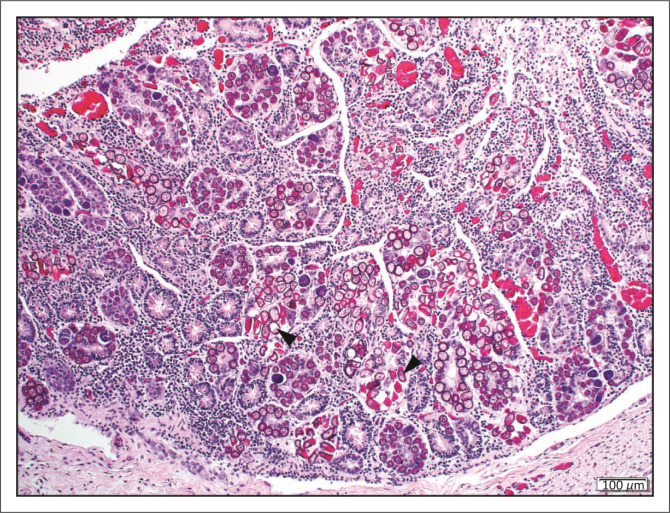
Intestinal coccidiosis. Many epithelial cells, predominantly within intestinal crypts, are distorted by intracytoplasmic coccidian organisms of various sexual stages including macrogametes, microgametes and oocysts (arrowheads) (haematoxylin and eosin [H&E]).

Eight impalas from GFs had intestinal coccidiosis associated with mild lymphoplasmacytic, eosinophilic and histiocytic enteritis (Figure 4). The coccidian burden varied considerably between individuals and within different intestinal segments of the same animal. One impala from a GF had crypt epithelial necrosis with mild villus blunting and fusion and low numbers of flagellated protozoa compatible with *Giardia* sp.

**FIGURE 5:**
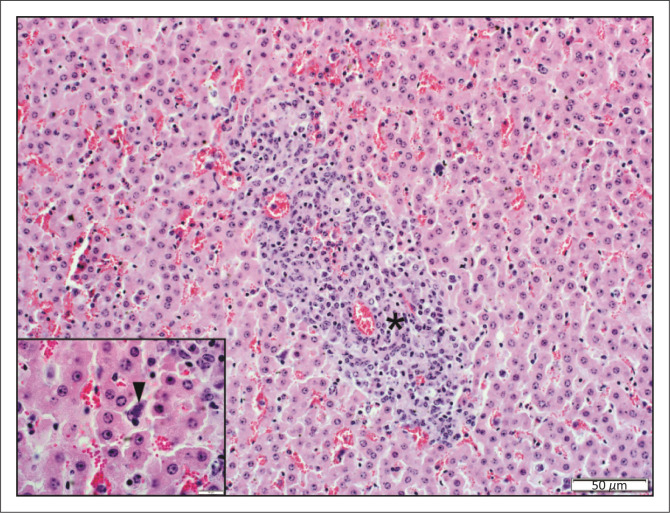
Hepatic theileriosis. Portal triad and surrounding sinusoids are infiltrated by large numbers of lymphoblastic cells (*) (haematoxylin and eosin [H&E]). Inset: Lymphoblastic cell (arrowhead) with intracytoplasmic schizont containing many 2–3 *μ*m diameter, crescent-shaped, basophilic merozoites.

Theileriosis was diagnosed in one juvenile, two adults and an impala of unspecified age, all from GFs. The lymph node, spleen, liver or lung showed perivascular infiltration by large mononuclear lymphoblastic cells, a few of which contained schizonts compatible with *Theileria* sp. (Figure 5). Confirmation of these by polymerase chain reaction (PCR) was not available.

### Infectious diseases

#### Bacterial infections

Animals with multicentric necrotising to suppurative inflammation, with or without concurrent intravascular bacteria and degenerate neutrophilic leucostasis within the pulmonary and splenic circulation, were diagnosed as having bacterial sepsis. Sepsis was diagnosed in 7% (17/251) of impalas but bacterial culture was only performed in seven cases. Mixed bacteria were often isolated, including *Escherichia coli, Streptococcus pneumoniae, Trueperella pyogenes*, and *Pasteurella multocida*. Two of the sepsis cases were neonates. Severe fibrinonecrotic omphalophlebitis and peritonitis were present in one neonatal animal from a zoo. In the other, sepsis was a consequence of aspiration pneumonia that may have resulted from recent tube-feeding. This lamb from a NP had been abandoned by its mother. It was suffering from brachygnathia inferior, hydrocephalus and cerebrocortical laminar necrosis of unknown aetiology. It also had extensive areas of epicardial, pericardial and cerebral mineralisation, which may have contributed to its death. No other tissues were affected and a cause for the mineralisation could not be determined. In one animal from a zoo, and another from a NP, the likely source of sepsis was post-partum endometritis. One septicaemic impala died during capture.

Two impalas from GFs and four from NPs had primary bacterial fibrinonecrotic and purulent bronchopneumonia with or without pleuritis. A monomorphic population of bacterial cocci was associated with the inflammation in a juvenile animal from a GF. Bacterial culture was not pursued and, as a result, the precise aetiology of the bronchopneumonia could not be determined. *Mycobacterium bovis* infection was diagnosed in an impala from a NP with granulomatous tracheobronchial lymphadenitis, pneumonia and splenitis with small numbers of intralesional acid-fast bacilli. Tuberculosis was confirmed by PCR for *M. tuberculosis* complex. Tissues of another animal from a NP tested positive on PCR for *M. tuberculosis* complex, but severe autolysis hindered detailed histopathological evaluation.

**FIGURE 6:**
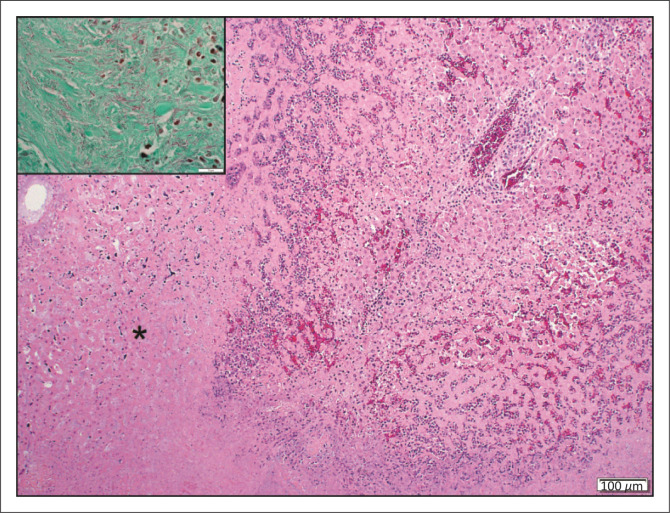
Hepatic necrobacillosis. A discrete focus of hepatic necrosis (*) surrounded by numerous degenerate neutrophils. Hepatic sinusoids within the adjacent viable parenchyma are congested (haematoxylin and eosin [H&E]). Inset: Myriad extracellular Gram-negative filamentous bacteria consistent with *Fusobacterium necrophorum* on the periphery of the necrotic region. Gram stain with cresyl green counterstain.

Impalas with tissues showing lytic and coagulative necrosis surrounded by numerous degenerate neutrophils and myriad extracellular filamentous gram-negative bacteria were diagnosed with necrobacillosis, although bacterial culture was not performed. Necrobacillosis (1%, 3/251) was uncommon and only seen in animals from GFs. One impala had ruminal necrobacillosis with subsequent spread to the liver; the tongue was affected in another animal (Figure 6, inset). The third impala had a 2-month history of inspiratory stridor with bilateral, severely thickened laryngeal arytenoid cartilages that resulted in partial obstruction of the laryngeal opening. Histologically, moderate focal necrotising neutrophilic laryngeal chondritis, with multiple irregular coalescing islands of disorganised cartilage and rare osteoid, was present, as well as severe subacute lymphoplasmacytic ulcerative laryngitis. No other lesions were noted in the tongue, oesophagus, trachea, heart or lungs (the only organs submitted).

Other bacterial infections were rare. Two impalas from GFs and one from a zoo had severe fibrinonecrotic, haemorrhagic and/or suppurative enteritis with large intralesional bacterial rods. *Clostridium perfringens* type A was cultured from the intestines of one of these impalas. In two animals from GFs, significant foci of hepatocellular degeneration and necrosis were associated with intralesional large bacilli that were morphologically consistent with *Clostridium* sp.

#### Viral infections

One juvenile impala, from a NP, had lesions consistent with FMD (including coronary band dermatitis with a large intraepithelial vesicle that contained sloughed epithelial cells), necrotic cellular debris and few neutrophils. However, definitive diagnosis could not be confirmed as viral isolation and immunohistochemistry were not available. Two impalas from NPs had cutaneous masses that were histologically consistent with cutaneous papillomas.

### Non-specific inflammatory lesions

Non-specific intestinal, hepatic, cutaneous, myocardial and renal inflammatory lesions could not be definitively linked to the above-mentioned infectious diseases. Splenic and lymph node reactive lymphoid follicular hyperplasia and lymph node medullary plasmacytosis and sinus histiocytosis were common (35%, 68/193), presumably as a response to infectious or parasitic disease. Impalas from NPs had significantly more (*p* < 0.0001) reactive change (72%, 44/61) than those from GFs (18%, 21/116) or zoos (15%, 2/13).

Mild to moderate eosinophilic and lymphoplasmacytic enteritis, with little or no associated epithelial damage, was seen in 31% (36/115) of impalas. Eight impalas (16%, 8/49) had neutrophilic to lymphoplasmacytic, periadnexal and perivascular dermatitis although no aetiological agents were detected. Dermatitis was not observed in impalas from zoos. Lymphoplasmacytic portal hepatitis with variable fibrosis and small bile duct proliferation, which variably extended beyond the limiting plate and into the surrounding parenchyma, was observed in 21% (41/198) of impalas from all three locations. Twenty-five (13%) impalas had mild multifocal, often perivascular, lymphoplasmacytic myocarditis without associated myocyte damage. Mild multifocal lymphoplasmacytic interstitial nephritis was observed in 8% (15/187) of impalas.

## Discussion

This study demonstrated the utility of histological databases for the characterisation and evaluation of lesions and mortality in wildlife species. However, given the inherent limitations of retrospective analysis, it does not provide a definitive assessment of disease prevalence within the entire impala population. Autolysis (particularly in tissues from impalas from NPs), incomplete tissue sampling, failure to perform ancillary diagnostic tests on fresh tissues and a paucity of historical data precluded complete diagnosis in some cases (14%, 34/251) and made other diagnoses often presumptive. A lack of viral isolation and/or molecular diagnostic techniques for non-domestic ruminants in South Africa also hampered full diagnostic and epidemiological investigations. Predation, limited surveillance and varying numbers of impala from the different locations likely influenced the apparent prevalence of disease, particularly in impalas from NPs. Diseases that are readily diagnosed by clinical examination or macroscopic pathology are likely under-represented in this study as these may not be submitted for histopathology examination (such as FMD in impalas from the KNP). Additional bias was likely introduced by the varying management of impalas in the three locations. Impalas from GFs form a continuum from intensively managed populations resembling zoos to largely unmanaged populations resembling NPs. Nonetheless, this study details a wide range of diseases and conditions seen in impalas and provides some indication of the likelihood of specific diseases occurring in impalas from GFs, NPs and zoos.

Non-infectious disease was the most common cause of morbidity and mortality. Acute pulmonary congestion and oedema, often associated with alveolar atelectasis and haemorrhage, were the most common histological finding. In the absence of significant inflammation or erythrophagia, pulmonary congestion and oedema were consistent with terminal shock or cardiopulmonary failure. This species may therefore be particularly prone to these terminal changes. Pulmonary congestion and oedema may have been less common in impalas from NPs because these animals were largely shot for tuberculosis monitoring purposes. In contrast, impalas from GFs or zoos mainly died naturally or were anaesthetised. Other terminal changes, which should not be misinterpreted as the cause of death, include aspiration of ruminal contents and drainage from areas of haemorrhage to regional lymph node sinuses.

Seven impalas died during or immediately following anaesthesia. Impalas are extremely sensitive to the respiratory suppressant effects of opioid capture drugs and can exhibit severe adverse reactions of sudden extreme respiratory depression and cessation of alveolar ventilation with generalised pulmonary oedema, alveolar collapse or atelectasis (Meyer et al. 2008; Zeiler & Meyer 2017). Concurrent pulmonary eosinophilic leucostasis, in two of these impalas, suggested that anaphylaxis, perhaps the result of anaesthetic drugs used, may have played a role in their deaths. As pulmonary congestion and oedema are not specific and can be readily observed as agonal change, a diagnosis of anaesthesia-associated respiratory failure must be based on the appropriate history and absence of other significant findings.

Over half of the animals with acute or subacute myocardial and/or skeletal muscle necrosis also had concurrent myoglobinuric nephrosis. Many of these impalas died during or after capture, and in the absence of other significant findings, acute muscle necrosis was thought to result from exertional (or capture) myopathy. In cases where myocardial necrosis was evident without significant skeletal muscle lesions, death may have been due to catecholamine release and stress associated with capture and handling (Meyer et al. 2008; Murray, Lewis & Coetzee 1981). Five over-conditioned, heavily pregnant animals that died immediately following capture during high ambient temperatures were thought to have died as a result of hyperthermia and exertional myopathy.

Cachexia was the second most common cause of death and was particularly common in impalas from GFs. Significant muscle mass and fat atrophy can be attributed to multiple factors, few of which were mentioned in the clinical histories provided. Possibilities include the provision of insufficient or poor-quality food due to overgrazing or drought conditions; limited access to food due to intra- and interspecific social factors, premature weaning or impaired mobility; maldigestion or malabsorption of food resulting from disease or parasitism in the gastrointestinal tract; or increased food requirements due to high levels of physical activity, the presence of disease or active reproductive status (Blanchard & Fritz 2008; Lane et al. 2014). The contribution of concurrent non-infectious, infectious and parasitic diseases to the cachexia is uncertain. In the majority of cachexic impalas, no other significant lesions were present; however, the quality and availability of food and social factors were unknown. As most wild ruminants avoid toxic plants, the presence of liver and neurological changes typically caused by the ingestion of toxic plants may indicate that cachexic impalas were kept in areas with poor grazing and/or insufficient browsing at the time of death. Tannins that occur naturally in tree leaves bind to and limit protein absorption and may contribute to cachexia in impalas and other browsers (Bothma et al. 2016). Cachexia may predispose individuals to increased mortality during stressful events like capture and environmental exposure, particularly in recently relocated animals that may not eat much in the first few days after relocation (Knox, Hattingh & Raath 1992; Zeiler & Meyer 2017). Cachexia may also have a negative effect on immune function, resulting in increased susceptibility to bacterial infections and/or parasitism (Kalantar-Zadeh et al. 2013). Old age and/or marked dental attrition contributed to cachexia in two impalas. One impala from a zoo had polyphasic muscle lesions similar to that caused by dietary deficiency of vitamin E and/or selenium in captive springbok (Mbatha et al. 2012). Ruminal acidosis is additional evidence of nutritional disease. Ruminal mineralisation was likely of no clinical consequence. The lesion is reportedly most common in impalas sampled in Southern African hot and cold seasons and, therefore, may be associated with the ingestion of significant dietary browse (Lane 1995). Based on these results, stocking rates, nutrition and pasture management on GFs may be adequate for large, dominant and commercially valuable species but not for the needs of smaller species such as impalas.

Traumatic injury was particularly common in impalas from zoos, perhaps because confinement, the use of multi-species enclosures and stocking densities collectively increase the likelihood of intra- and interspecies aggression and collision with enclosure walls or entanglement in fences. Lymphoid depletion was most commonly observed in impalas from zoos. Most were neonates; neonatal impalas from the other locations may have been under-represented due to less intense animal surveillance and predation. Lymphoid depletion is a non-specific finding that can be stress-related (Gruver & Sempowski 2008).

Lesions that were presumed to result from toxins were uncommon and included hepatic lesions consistent with those caused by aflatoxin or plant toxins such as *Senecio* and *Lantana* spp. and neurological lesions associated with *Trachyandra* or *Phalaris* spp. in domestic ruminants (Kellerman et al. 2005). Concurrent ulcerative dermatitis of the external pinna in one of these cases likely resulted from type III photosensitivity (Kellerman et al. 2005). An impala from a NP, with lesions compatible with intoxication by microcystin (Bengis et al. 2016), indicates that anthropogenic factors can influence the health of all three populations. Neoplasia was extremely rare and considered of no clinical significance when present.

Impalas from NPs had the highest prevalence of endoparasitism and ectoparasite-related dermatitis. This finding was not unexpected as captive animals may undergo deworming and acaricide treatment and may not have contact with necessary intermediate hosts. Parasitic lung and liver lesions are common amongst impalas (Gallivan et al. 1996, 1989; Pletcher et al. 1988; Van Wyk & Boomker 2011). Whilst they can be macroscopically misinterpreted as tuberculosis, the histological appearance is usually diagnostic so acid-fast stains, *Mycobacterium* sp. A PCR or culture is rarely required. Ectoparasite infestation is particularly high in periods of nutritional stress such as during droughts or in the late dry season (August – October) (Horak et al. 2003), or in impalas from areas with warm wet climates (Gallivan & Surgeoner 1995). Lymphoid hyperplasia and other features of reactive change were significantly more common amongst animals from NPs, perhaps due to the high parasite burden in this population. The importance of ectoparasite control in captive impala populations is highlighted by the absence of theileriosis in zoo animals; and by the disease being present in one impala that was recently relocated from an area where the disease was not present to an area in which theileriosis was endemic.

Myocardial and skeletal muscle sarcocystosis, although very common, was considered incidental. Sarcocysts were morphologically consistent with *Sarcocystis melampi* previously reported in impalas (Junker, Horak & Penzhorn 2015). The life cycle of *Sarcocystis sp.* in many wildlife species is not documented; the presence of the protozoa in herbivore tissues indicates carnivore faecal contamination of herbivore food. Intestinal coccidiosis was detected in eight impalas. *Eimeria* sp. infection has been previously reported in impalas (Junker et al. 2015) and control may be facilitated, as in intensive domestic animal production systems, by raising water troughs off the ground and preventing run-off from middens into water sources. No cryptosporidiosis was diagnosed although the parasite has been recorded in impalas (Abu Samra et al. 2013).

Only 11% (27/251) of impalas had lesions compatible with specific infectious diseases, although minor non-specific inflammation was present in many tissues. The low prevalence of tuberculosis and FMD was similar to the results of previous studies (Clifford et al. 2013; Okori 1999). Only two impalas were diagnosed with tuberculosis, despite the submission of multiple animals from the Greater KNP area, in which bovine tuberculosis is endemic (Bengis 2000). Nonetheless, the true prevalence of the infection in impalas is unknown. Two impalas from NPs had cutaneous papillomatosis-resembling lesions described in other South African antelope, from which bovine papilloma viruses have been identified (Williams et al. 2011). One impala from a GF had intestinal lesions suggestive of a viral infection, but the cause was not determined. The neonatal impala from a NP with hydrocephalus and brachygnathia also had splenic and lymph node lymphoid depletion; consequently, its death due to sepsis may have been facilitated by immune suppression and/or stress. The animal had been abandoned by its mother, perhaps due to congenital lesions and foetal ischemia during birth. It is uncertain if the congenital abnormalities were a sporadic congenital condition or perhaps induced by viral infection such as the Akabane virus, to which impalas are known to seroconvert (Al-Busaidy, Hamblin & Taylor 1987). Theileriosis and necrobacillosis were rare and only affected animals from GFs. Immune compromise may have increased the susceptibility of these animals to the *Theileria* sp. infection (Mans, Pienaar & Latif 2015). Cachexia, malnutrition and/or stress due to overcrowding, human interference or inbreeding may therefore influence disease prevalence in captive impalas. Insufficient numbers of impala with a variant coat colour were available to assess whether or not selection for such variants had any implications for the animal’s health and immune status.

Interestingly, the impala has been unchanged in its basic form for at least the past 5 million years. In contrast, the alcelaphine common ancestor has diverged at least 18 times during the same time period, into various forms of hartebeest and wildebeest (Fritz & Bourgarel 2013). Impalas may therefore be efficiently adapted to their environment with no selection for major changes in morphology or behaviour since prehistoric times. The animal’s gregarious nature, facultative browsing feeding strategy, and flexible habitat usage and territorial systems may play a role in the apparent resistance of impala to infectious disease (Bothma et al. 2016; Skinner & Chimimba 2005).

## Conclusion

Despite the limitations of this retrospective review, it showed that management and environmental factors significantly impact disease in impalas on GFs. This study suggests that nutrition and pasture management, enclosure design, management of intra- and interspecies aggression and improved anaesthetic protocols could improve animal welfare and the survival of impalas on GFs and in zoos. Although it appears that impalas seldom succumb to infectious diseases, they commonly show mild, non-specific inflammatory lesions and may act as potential reservoir hosts for a variety of diseases that may be detrimental to other species. Continued surveillance and more detailed disease investigation are important for the continued health of the impala population, whether in zoos, GFs or NPs.
